# A new genus of leafhopper subtribe Paraboloponina (Hemiptera: Cicadellidae) with molecular phylogeny of related genera

**DOI:** 10.1371/journal.pone.0177644

**Published:** 2017-05-24

**Authors:** Naresh M. Meshram, Pathour R. Shashank, Twinkle Sinha

**Affiliations:** National Pusa Collection, Division of Entomology, ICAR-Indian Agricultural Research Institute, New Delhi, INDIA; Sichuan University, CHINA

## Abstract

A new leafhopper genus *Chandra* and species *Chandra dehradunensis* **gen. nov., sp. nov**. are described, illustrated from India and placed in the subtribe Paraboloponina (Cidadellidae: Deltocephalinae: Drabescini). This genus is closely associated with the genus *Parabolopona* Webb but differs in shape of the head, placement of antennae, male genitalia and molecular analysis using Histone *H3* and *COI* genes confirmed the difference. The taxonomic and phylogenetic position of *Chandra* is discussed using morphological characters and preliminary molecular evidence of the new genus and related genus *Parabolopona*.

## Introduction

Paraboloponini, primarily distinguished by the striated fore margin of the head and long antennae situated near middle to upper corners of eyes, was subsequently placed as a subtribe Paraboloponina of Drabescini of Deltocephalinae by Dmitriev [1] and Zahniser and Dietrich [2,3]. Zahniser and Dietrich [3] revised the classification of Deltocephalinae based on the molecular and morphological data and provided a revised interpretation of Drabescini with two subtribe Drabescina and Paraboloponina, including 38 genera, out of which 36 genera belong to Paraboloponina with 132 species worldwide. Paraboloponina can be distinguished from Drabescina by the antennal ledges which are weak or absent, antennae usually longer, 1.5x width of head or longer, texture of the frontoclypeus shagreen, protibia rounded dorsally or rarely somewhat flattened, and forewing appendix broad [3].

Work on this group since Zhang and Webb [4], Webb [5], Viraktamath [6] and Meshram, et al. [7] has lead to description of many new taxa. So far, Paraboloponina contains 14 known genera, including 37 species from India. In the present work we describe a new leafhopper genus and species *Chandra dehradunensis* gen. nov. and sp. nov., and discuss its phylogenetic position within Drabescini, as inferred from available molecular data.

## Materials and methods

### Collection of samples and morphological study

Collections were not done from any national park or other protected area of land or sea, or on any private land, hence no permission was required. No specific permissions were required for any of the collection localities/activities, as the collections were done in and around ICAR research Institutes. The field studies did not involve any endangered or protected species

Specimens were collected through Mercury vapour lamp light trap from Dehradun (India: Himachal Pradesh), were processed by series of steps like sorting, cleaning and mounting.

Male genitalia dissection was carried out as described by Oman [8] and Knight [9]. The abdomen was removed by inserting a sharp pin between the abdomen and thorax with gentle piercing. The abdomen was treated in 10% KOH for 2~4 h to remove unsclerotized material by gently prodding the abdomen with the head of a pin. Afterwards, the abdomen was rinsed thoroughly in water. The internal structures were then removed by a hooked pin, before being stored in glycerol vials for study.

Photographs were taken with a Leica DFC 425C digital camera on the Leica M205FA stereozoom automontage microscope.

### Molecular study

#### DNA extraction and PCR amplification

For Histone*H3* and mitochondrial cytochrome oxidase subunit I (mt*COI*) analysis, the DNA was extracted from legs of specimens according to the manufacturer protocols, QIAGEN QIAamp^®^ DNA Investigator Kit. The isolated DNA was stored at -20°C until required [10]. The DNA extractions were amplified for PCR products, Histone H3 primers are: HEXAF (forward) 5'-ATGGCTCGTACCAAGCAGACGGC-3' and HEX- AR (reverse) 5'-ATATCCTTGGGCATGATGGTGAC-3' [11] mtCOI primers are LCO1490: 5’-GGTCAACAAATCATAAAGATATTGG-3’; HCO2198: 5’-TAAACTTCAGGGTGACCAAAAAATCA-3’ [12]. The PCR protocol for Hisone H3 followed Zahniser and Dietrich (2010) and mtCOI was amplified in 25 μl reactions using DNA polymerase (Fermentas GmBH, St. Leon- Rot, Germany) under the following cycling protocol: 4 min. hot start at 94°C, 35 cycles of denaturation for 30 s at 94°C, annealing for 60 s at 47°C, elongation for 50 s at 72°C and a final extension 72°C for 8min in a C1000^™^ Thermal cycler. The reactions were combined (as described by KOD FX puregene^™^ manufacturer protocol) of DNA template 4 μl, 2x PCR buffer 12.5 μl, 2mM dNTP 10 μl, TAQ (KODFX) enzyme 1 unit, and forward and reverse primers were 0.3 μM each at final concentration. The products were checked on 2% agarose gel and visualized under UV using Alphaview^®^ software version1.2.0.1. The amplified products were sequenced at SciGenome Pvt. Ltd. (Cochin, India). The quality sequences were assembled with BioEdit version 7.0.0 and deposited in NCBI GenBank.

#### Molecular phylogenetic analyses

Histone H3 and COI sequences in FASTA format were imported into the sequence alignment application of MEGA 6.05 [13] software package and multiple sequence alignments were performed with the ClustalW [14] algorithm using default parameters. The Basic Local Alignment Search Tool (BLAST) [15] was used to query the National Center for Biotechnology Information (NCBI) non-redundant nucleotide database and protein database with other leafhopper Histone H3 and COI sequence data in blastn and blastx searches, respectively. The sequences were submitted to NCBI for GenBank Accessions (Tables 1 and 2).

**Table 1:** Showing GenBank Accession Numbers, along with locality data for Histone H3.

Species	Geographic locality	GenBank sequence ID
*Drabescus sp*.	Taiwan: Taipei Co.	GU123824*
*Scaphoidophyes* nr. *pyrus*	Zambia: Copperbelt Prov.	JX845554*
*Xestocephalus desertorum*	USA: Illinois	GU123892*
*Phlogotettix cyclops*	Taiwan: Ilan Prov.	GU123874*
*Bhatia satsumensis*	Taiwan: Taipei Co.	GU123803*
*Parabolopona guttata*	Taiwan: Nantou Co.	GU123866*
*Chandra dehradunensis* gen. nov., sp. nov.	India: Himachal Pradesh	KY496185
*Chandra dehradunensis* gen. nov., sp. nov.	India: Himachal Pradesh	KY496186
*Parabolopona zhangi*	India: Meghalaya	KY496187
*Parabolopona zhangi*	India: Meghalaya	KY496188
*Parabolopona zhangi*	India: Meghalaya	KY496189

*Downloaded from Genbank

**Table 2:** Showing GenBank Accession Numbers, along with locality data for mtCOI.

Species	Geographic locality	Genebank sequence ID
*Parabolopona zhangi*	India: Meghalaya	KU535896*
*Scaphoideus carinatus*	Canada: Quebec, La Mauricie National Park	KR343386*
*Osbornellus auronitens*	Canada: Ontario, Rouge National Urban Park	KR578363*
*Phlogotettix sp*.	India: Mizoram:	KM047668*
*Mimotettix sp*	Not available	JX433212*
*Aphrodes diminuta*	Canada: Ontario, Leeds and Grenville	KF321754*
*Chandra dehradunensis* gen. nov. *sp*. *nov*.	India: Himachal Pradesh	KY496190
*Chandra dehradunensis* gen. nov. *sp*. *nov*.	India: Himachal Pradesh	KY496191
*Parabolopona zhangi*	India: Meghalaya	KY496192

*Downloaded from Genbank

Sequence divergences between selected leafhoppers were calculated using the Kimura 2-Parameter distance model [16] and graphically displayed in a neighbor-joining (NJ) tree [17] by the program MEGA 6.06 [13]. Tree robustness was evaluated by bootstrapping (Felsenstein, 1985) with 2,000 replicates with the *Xestocephalus desertorum* for Histone H3 and *Aphrodes diminuta* for COI sequence as outliers.

### Nomenclatural acts

The electronic edition of this article conforms to the requirements of the amended International Code of Zoological Nomenclature, and hence the new names contained herein are available under that Code from the electronic edition of this article. This published work and the nomenclatural acts it contains have been registered in ZooBank, the online registration system for the ICZN. The ZooBank LSIDs (Life Science Identifiers) can be resolved and the associated information viewed through any standard web browser by appending the LSID to the prefix “http://zoobank.org/”. The LSID for this publication is: urn:lsid:zoobank.org:pub:FAF956DF-D6C5-4153-85D5-8F2ACD572260. The electronic edition of this work was published in a journal with an ISSN, and has been archived and is available from the following digital repositories: PubMed Central, LOCKSS.

### Depositories of material

Type material is deposited in the National Pusa Collection, Division of Entomology, Indian Agricultural Research Institute, New Delhi, (NPC) and University of Agricultural Sciences Bangalore (USAB), India.

## Results and discussion

### Taxonomy

#### *Chandra* Meshram gen. nov.

**Fig 1:**
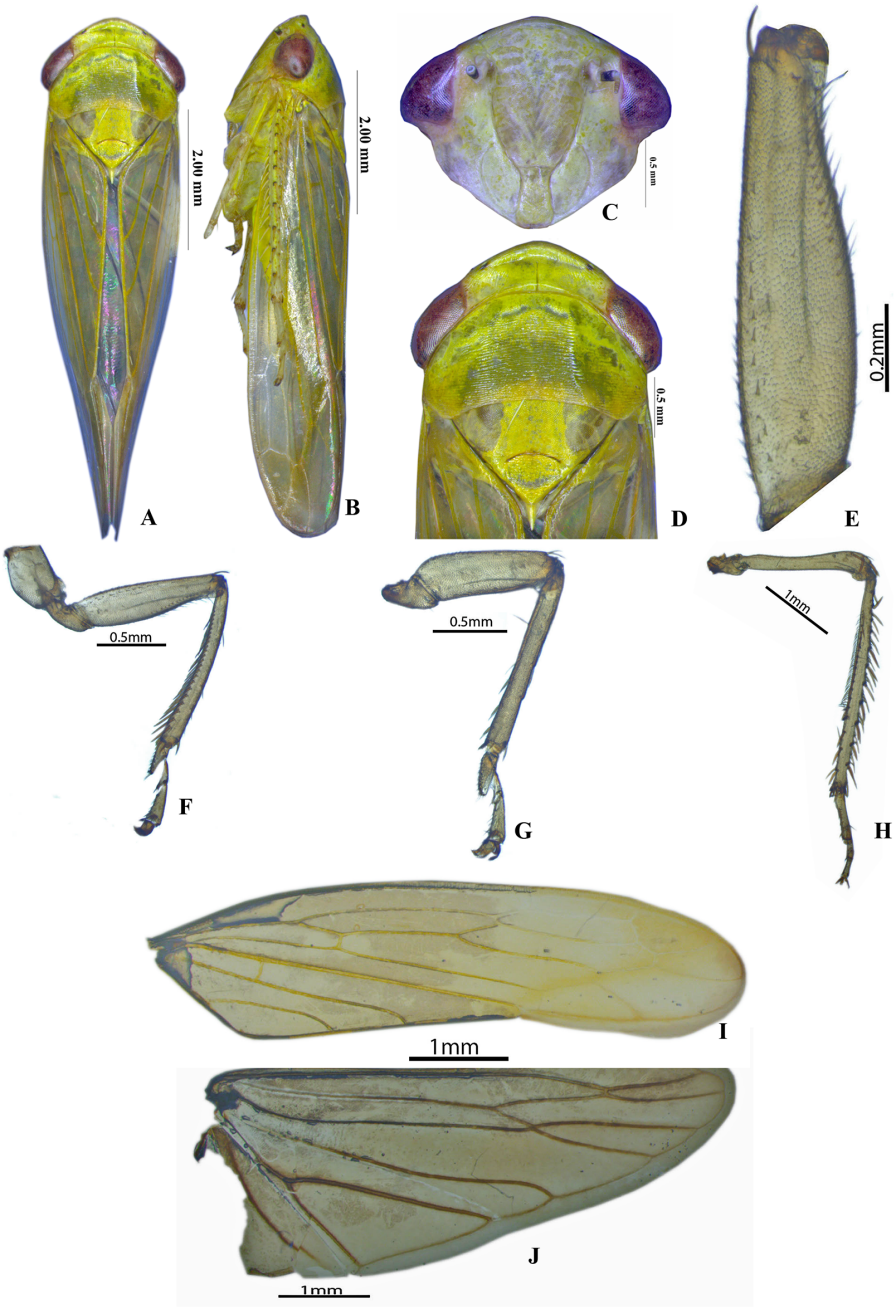
*Chandra dehradunensis* Meshram gen. nov., sp. nov., male A-B. Habitus, dorsal and lateral view; C Face; D. Head and Pronotum; E. Prothoracic femur; F. Prothoacic leg; G. Mesothoacic leg, H. Meatathoraic leg; I. Forewing; J. Hind wing.

**Fig 2:**
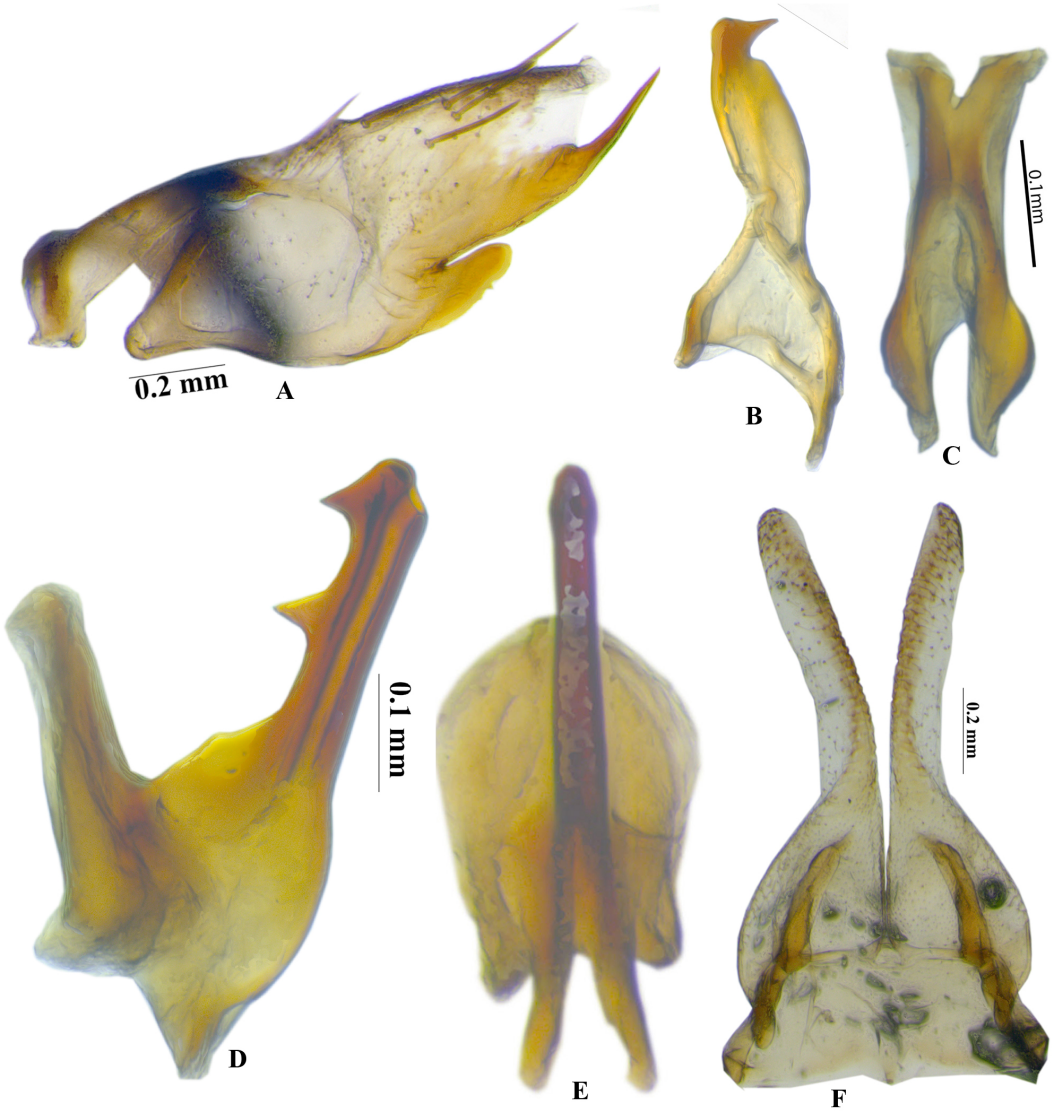
*Chandra dehradunensis* Meshram gen. nov., sp. nov., male genitalia A. Pygofer; B. Style; C. Connective; D-E. Aedeagus, lateral and dorsal view; F. Subgenital plate.

urn:lsid:zoobank.org:act:9F07D9A6-C112-41E2-9A85-8C94C847A236 (Figs 1 and 2) Type Species: ***Chandra dehradunensis* sp. nov. Meshram**

#### Diagnosis

Yellow to green; vertex green, face pale. Scutellum medially with bright yellow band, laterally surrounded by two translucent triangles below hind margin of pronotum. Forewing pale green tinged with greenish yellow, veins yellowish having few brown spots (Fig 1A–1D).

Male: Head in dorsal view 3x wider than pronotum. Crown 3x broader than its length anterior and posterior margins almost parallel; texture smooth with distinct transverse depression preapically; ocelli small, on crown just posterad of anterior margin, mesad of antennal pits and well separated from eyes (Fig 1C–1D). Frontoclypeus, not tumid, smooth, in anterior view evenly broadened from anteclypeus to dorsal margin. Clypellus widening apically, apex following or slightly surpassing normal curve of gena. Lorum subequal to or wider than clypellus near base. Antennal ledges weakly developed, Antennal bases near upper or anterodorsal corners of eyes. Antennal pits often very large and encroaching onto frontoclypeus. Antennae long, 1.5 x width of head or longer. Gena broad not incised (Fig 1C).

Pronotum depressed, 2x as broad as long, anterior margin produced but not extended anterad of eyes; posterior margin weakly concave, transverse striae well developed, lateral margin shorter than eye, long, carina present, curved dorsally (Fig 1D). Proepisternum obscure. Scutellum1.3x broader than its width and 1.2x longer than pronotum, with transverse depression distinct; area distad of transverse depression distinctly elevated posteriorly, without sharp lateral edge (Fig 1B–1D). Forewing elongate; four apical cells; outer and middle subapical cell closed; inner subapical cell open; two or three cross veins present between claval veins; claval margin not elevated, apex of clavus weakly crimped; appendix extended around apex (Fig 1I). Hindwing venation complete; m-cu crossvein subapical (Fig 1J). Prothoracic femur, with AM setae present, AV setae long, hairlike, AD setae small and sparse (Fig 1E). Prothoracic tibia with dorsal surface rounded but not expanded (Fig 1F); AD and PV setae sparse; PD setae very dense; AV setae moderately dense and long (Fig 1F). Mesothoracic femur with AD1and PD1 setae. Metathoracic femur with setal formula 2+1+1(Fig 1G); lateral surface broadened distally with dense irregularly arranged setae distally (Fig 1G). Metathoracic tibia flattened, tibial row PD with long macrosetae, AD with macrosetal bases spinelike, AV with macrosetae, PV with numerous long tapered setae, tarsomere II less than 1/2 length of tarsomere I (Fig 1H).

Male pygofer short; in lateral view dorsal margin slightly convex (Fig 2A); posteroventral margins with spine-like ventral pygofer process arising somewhat in the middle, below which small digitate lobe arises (Fig 2A); several macrosetae and near dorsal and distal margin (Fig 2A). Subgenital plate tapering from mid-posteriorly to membranous fingerlike, glabrous process; slightly shorter than pygofer (Fig 2F). Connective Y-shaped, stem and arms subequal (Fig 2C). Style broadly bilobed basally, median anterior lobe pronounced (Fig 2B). Aedeagus V-shaped with well-developed dorsal apodeme, this 0.75 as long as shaft, dorsal margin of shaft with pair of apical and middle teeth, gonopore apical (Fig 2D–2E).

Female: Unknown.

#### Etymology

The genus is named after Prof. Chandrashekhara A. Viraktamath in recognition of his monumental contributions to leafhopper taxonomy.

#### Distribution

Himachal Pradesh, India.

#### Remarks

This genus can be distinguished from its closely associated genus *Parabolopona* by the following combination of characters: vertex strongly concave (Fig 1D); Prothoracic femur, with AM setae present (Fig 1E); Mesothoracic femur with AD1and PD1 setae; posteroventral margins of pygofer with spine like ventral pygofer process arising somewhat in the middle, below which small digitate lobe arises (Fig 1G); Subgenital plate tapering from mid-posteriorly to membranous fingerlike, glabrous (Fig 2F); Aedeagal shaft with pair of apical and middleteeth (Fig 2D–2E).

#### *Chandra dehradunensis* Meshram sp. nov.

urn:lsid:zoobank.org:act:31C31F1A-6F77-4F84-933A-A27B44D9D6A9 (Figs 1 and 2)

#### Diagnosis

External morphology, as in generic description. Male genitalia, as in generic description, pygofer with lobe (Fig 2A) broadly truncate in lateral view. Connective Y-shaped, stem and arms subequal (Fig 2C). Style with beak-like apophysis (Fig 2B). Aedeagal shaft with pair of apical and middle teeth (Fig 2D).

#### Female

Unknown.

#### Type material

Holotype ♂, INDIA: Uttarakhand: Dehradun: Muktapakhari: New Chakrata, (30.3165° N, 78.0322° E), 16.vi.2015, Mercury vapour lamp Coll. Rahul Chaubey (NPC). Paratypes 4♂ with same data as on holotype.

#### Etymology

The species was named after the place of collection Dehradun in Uttarakhand India.

## Discussion

### Taxonomic position

**Fig 3:**
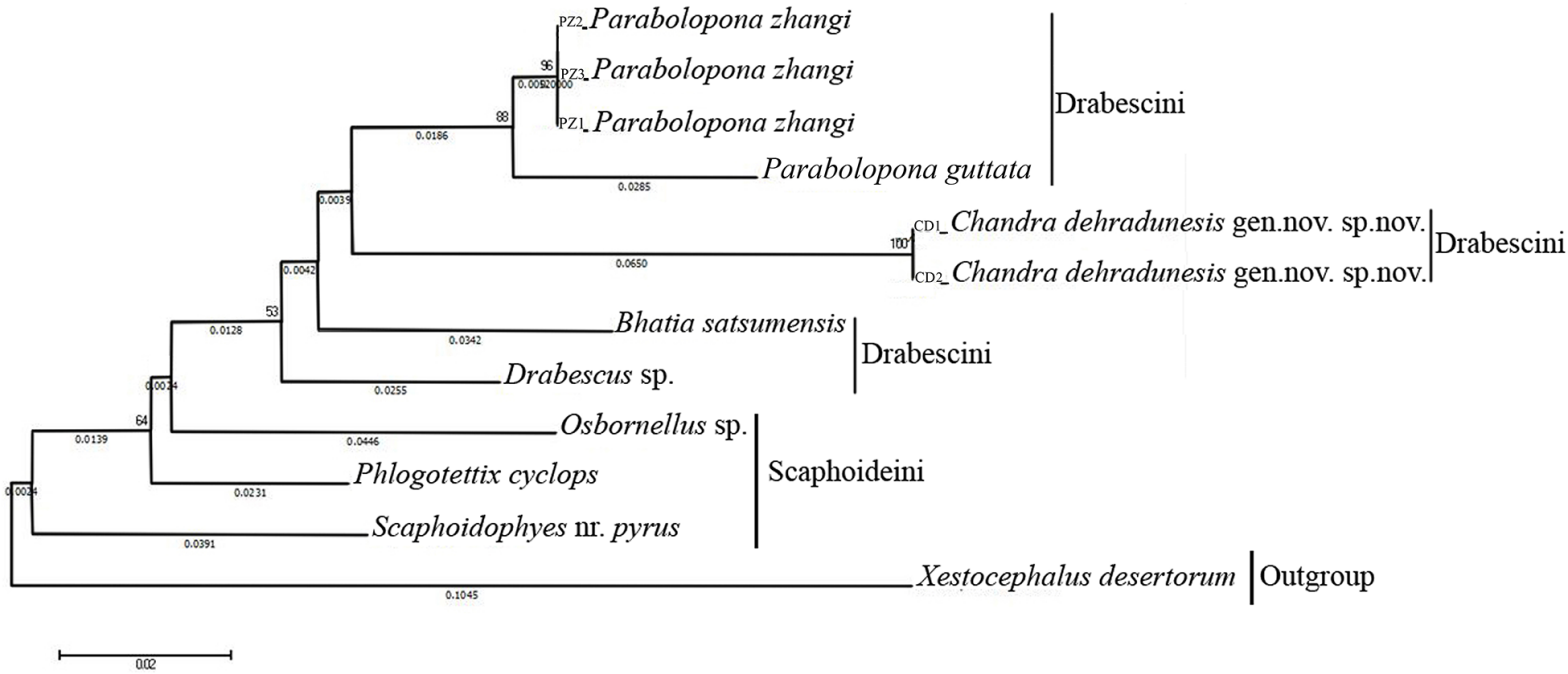
Phylogram of the analysed genera Deltocephalinae showing relationships of *Chandra dehradunensis* Meshram gen. nov., sp. nov. with related genera of tribe drabescini inferred using by neighbor-joining (NJ) tree method and the kimura 2-parameter distances of Histone H3 sequences.

**Fig 4:**
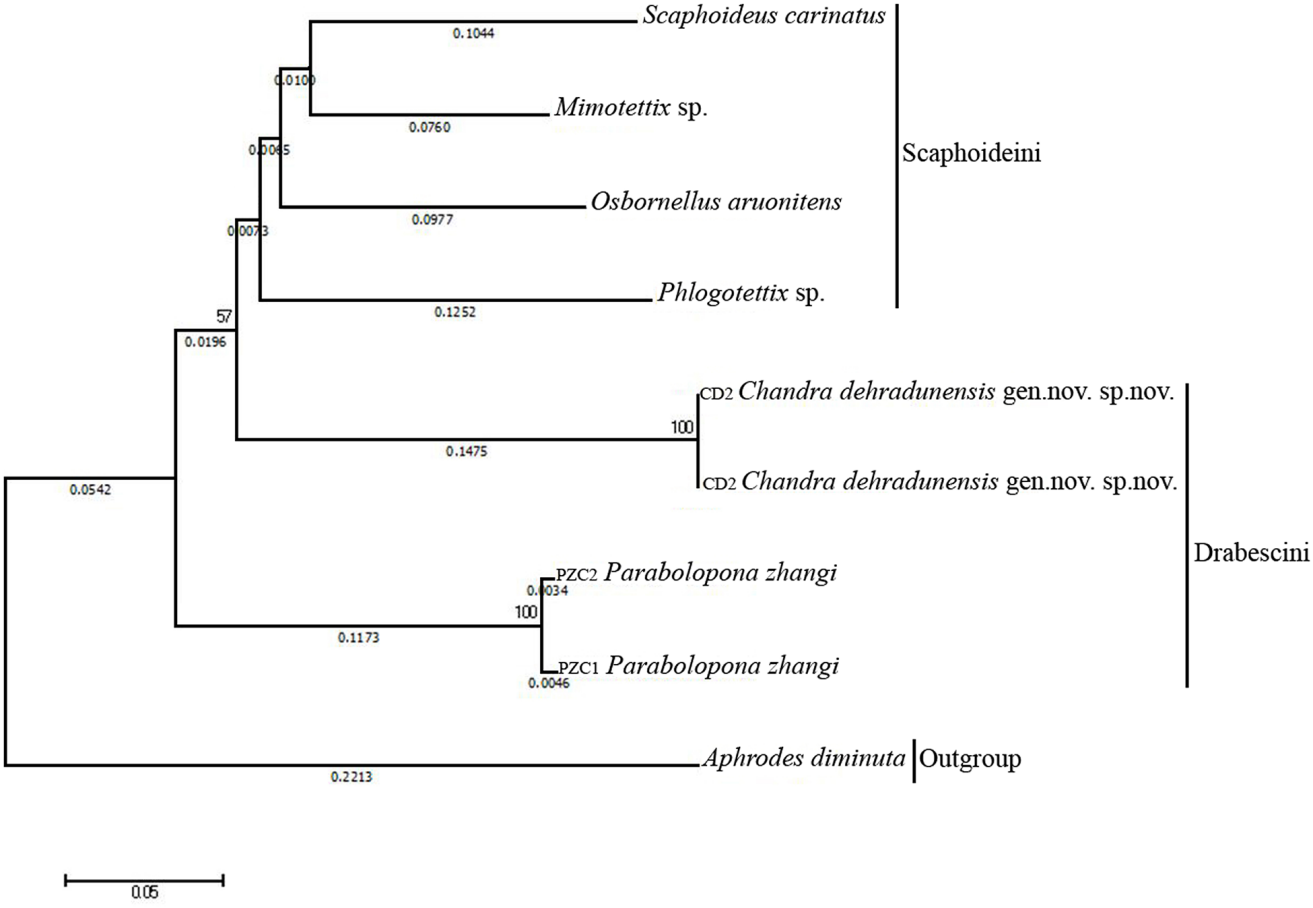
Phylogram of the analysed genera Deltocephalinae showing relationships of *Chandra dehradunensis* Meshram gen. nov., sp. nov. with related species of tribe drabescini inferred using by neighbor-joining (NJ) tree method and the kimura 2-parameter distances mitochondrial COI sequences.

The systematic position of *Chandra dehradunensis* Meshram gen. nov. and sp. nov. as suggested by morphological evidence due to the presence of a peculiar combination of characters of tribe Drabescini. This genus can be distinguished from its closely associated genus *Parabolopona* by the following combination of characters: vertex in dorsal view strongly concave (Fig 1D); prothoracic femur, with AM setae present (Fig 1E); mesothoracic femur with AD1and PD1 setae; posteroventral margins of pygofer with spine like ventral pygofer process arising somewhat in the middle, below which small digitate lobe present (Fig 1G); subgenital plate tapering from mid-posteriorly to membranous, fingerlike, glabrous (Fig 2F); aedeagal shaft with pair of apical and middle (Fig 2D–2E). Most of these characters also separate *Chandra* gen. nov. from other known deltocephaline groups. Our preliminary molecular analysis using Histone H3 gene and Cytochromne oxidase I (Figs 3 and 4) agrees with the morphological and biogeographic phylogeny of Zahniser and Dietrich (2013) and places them in the subtribe Paraboloponina of tribe Drabescini.

To test the actual phylogenetic position of the new genus in the context of the morphologically related genera of Drabescini, we performed a preliminary molecular analysis using available material of a series of taxa within Drabescini from NCBI GenBank (Table 1). Histone H3 analysis resulted to be the most closely (*Parabolopona*, *Bhatia*, *Drabescus* and *Osbonellus*) or the most distantly related (*Phlogotettix*, *Scaphoidophyses* and *Xestocephalus*) (S1 Table). The final data matrix of our preliminary phylogenetic analysis (Table 1) included 11 terminals (10 ingroup taxa belonging to 4 genera of Deltocephalinae and 1 outgroup taxon). The availability of mtCOI sequence information is very limited for species of Drabescini. Due to this reason we have selected *Chandra* gen. nov., *Parabolopona*, *Scaphoideus*, *Osbornellus*, *Phlogotettix*, *Mimotettix* and *Aphrodes* (Table 2) for phylogenetic analysis (S2 Table).

Analysis of Histone H3 revealed that the sequence variation between new genus and *Parabolopona* 8.80 percent (S1 Table). The other closest genera are *Bhatia*, *Drabescus* and *Osbonellus*, all these four genera will share the same tribe (Drebescini) and two of them *Parabolopona*, *Bhatia* sharing same subtribe (Paraboloponina). Therefore, molecular data complement the morphological data of placing new genus into Paraboloponina. Furthermore, analysis of COI data places new genus with *Parabolopona zhangi* and consistent with Histone *H3* analysis.

## Conclusions

The combined morphological and molecular analysis clearly indicate and confirm that *Chandra dehradunensis* Meshram gen. nov., sp. nov., is near the distinct genus *Parabolopona*. Our molecular analyses also suggest that there is a large variation between new genus and other related genera of tribe Drabescini. Phylogeny estimated from the molecular data for new genus follows a previous phylogeny for subfamily Deltocephalinae from Zahniser and Dietrich (2013) and places *Chandra dehradunensis* gen. nov., sp. nov. in the subtribe Paraboloponina. There is a need for further detailed study using more molecular data to establish intricacies within the tribe Drabescini.

## Supporting information

S1 Table Percent pairwise corrected (K2P) genetic distance among different species of Deltocephalinae including the new genus for Histone H3. (DOCX)

S2 Table Percent pairwise corrected (K2P) genetic distance among different species of Deltocephalinae including the new genus for MtCOI. (DOCX)
