# Supplementary material for: A new genus of leafhopper subtribe Paraboloponina (Hemiptera: Cicadellidae) with molecular phylogeny of related genera
Source: PLoS One. 2017 May 24;12(5):e0177644. doi: 10.1371/journal.pone.0177644 (PMC5443518; doi:10.1371/journal.pone.0177644)
Supplement: S1 Table — (DOCX) [file pone.0177644.s001.docx]

**Supplementary Table 1. Percent pairwise corrected (K2P) genetic distance among different species of Deltocephalinae including the new genus for Histone H3**

| **Species** | **1** | **2** | **3** | **4** | **5** | **6** | **7** | **8** | **9** | **10** | **11** | **12** |
| --- | --- | --- | --- | --- | --- | --- | --- | --- | --- | --- | --- | --- |
| *Scaphoidophyes_nr._pyrus* |  | 0.024 | 0.018 | 0.022 | 0.028 | 0.025 | 0.022 | 0.025 | 0.025 | 0.022 | 0.022 | 0.022 |
| *Osbornellus_sp.* | 0.111 |  | 0.018 | 0.018 | 0.029 | 0.020 | 0.024 | 0.026 | 0.026 | 0.021 | 0.021 | 0.021 |
| *Phlogotettix_cyclops* | 0.074 | 0.070 |  | 0.018 | 0.027 | 0.021 | 0.018 | 0.021 | 0.021 | 0.016 | 0.016 | 0.016 |
| *Drabescus_sp.* | 0.101 | 0.074 | 0.074 |  | 0.030 | 0.016 | 0.019 | 0.022 | 0.022 | 0.015 | 0.015 | 0.015 |
| *Xestocephalus_desertorum* | 0.146 | 0.157 | 0.146 | 0.167 |  | 0.029 | 0.030 | 0.037 | 0.037 | 0.033 | 0.033 | 0.033 |
| *Bhatia_satsumensis* | 0.125 | 0.087 | 0.101 | 0.062 | 0.161 |  | 0.019 | 0.022 | 0.022 | 0.016 | 0.016 | 0.016 |
| *Parabolopona_guttata* | 0.106 | 0.115 | 0.074 | 0.079 | 0.157 | 0.083 |  | 0.024 | 0.024 | 0.011 | 0.011 | 0.011 |
| CD1_*Chandra_dehradunensis__gen._nov.,_sp._nov.* | 0.121 | 0.124 | 0.096 | 0.102 | 0.213 | 0.106 | 0.116 |  | 0.000 | 0.020 | 0.020 | 0.020 |
| CD2_*Chandra_dehradunensis__gen._nov.,_sp._nov.* | 0.121 | 0.124 | 0.096 | 0.102 | 0.213 | 0.106 | 0.116 | 0.000 |  | 0.020 | 0.020 | 0.020 |
| PZ1_*Parabolopona_zhangi* | 0.101 | 0.096 | 0.070 | 0.058 | 0.184 | 0.062 | 0.034 | 0.088 | 0.088 |  | 0.000 | 0.000 |
| *PZ2_Parabolopona_zhangi* | 0.101 | 0.096 | 0.070 | 0.058 | 0.184 | 0.062 | 0.034 | 0.088 | 0.088 | 0.000 |  | 0.000 |
| *PZ3_Parabolopona_zhangi* | 0.101 | 0.096 | 0.070 | 0.058 | 0.184 | 0.062 | 0.034 | 0.088 | 0.088 | 0.000 | 0.000 |  |
