# Supplementary material for: A new genus of leafhopper subtribe Paraboloponina (Hemiptera: Cicadellidae) with molecular phylogeny of related genera
Source: PLoS One. 2017 May 24;12(5):e0177644. doi: 10.1371/journal.pone.0177644 (PMC5443518; doi:10.1371/journal.pone.0177644)
Supplement: S2 Table — (DOCX) [file pone.0177644.s002.docx]

**Supplementary Table 2. Percent pairwise corrected (K2P) genetic distance among different species of Deltocephalinae including the new genus for MtCOI**

| **Species** | **1** | **2** | **3** | **4** | **5** | **6** | **7** | **8** | **9** |
| --- | --- | --- | --- | --- | --- | --- | --- | --- | --- |
| CD1_*Chandra_dehradunensis__gen._nov.,_sp._nov.* |  | 0.000 | 0.033 | 0.033 | 0.032 | 0.034 | 0.029 | 0.034 | 0.053 |
| CD2_*Chandra_dehradunensis__gen._nov.,_sp._nov.* | 0.000 |  | 0.033 | 0.033 | 0.032 | 0.034 | 0.029 | 0.034 | 0.053 |
| PZC2_*Parabolopona_zhangi* | 0.273 | 0.273 |  | 0.031 | 0.031 | 0.032 | 0.031 | 0.004 | 0.047 |
| *Scaphoideus_carinatus* | 0.267 | 0.267 | 0.264 |  | 0.029 | 0.028 | 0.024 | 0.032 | 0.048 |
| *Osbornellus_auronitens* | 0.262 | 0.262 | 0.258 | 0.224 |  | 0.028 | 0.022 | 0.032 | 0.049 |
| *Phlogotettix_sp* | 0.283 | 0.283 | 0.263 | 0.240 | 0.224 |  | 0.029 | 0.032 | 0.048 |
| *Mimotettix_sp* | 0.242 | 0.242 | 0.256 | 0.180 | 0.171 | 0.235 |  | 0.031 | 0.041 |
| PZC1_*Parabolopona_zhangi* | 0.282 | 0.282 | 0.008 | 0.264 | 0.258 | 0.264 | 0.256 |  | 0.047 |
| *Aphrodes_diminuta* | 0.453 | 0.453 | 0.399 | 0.422 | 0.416 | 0.431 | 0.369 | 0.395 |  |
